# Decoding Caregiver Burden in Cancer: Role of Emotional Health, Rumination, and Coping Mechanisms

**DOI:** 10.3390/healthcare11192700

**Published:** 2023-10-09

**Authors:** Ipek Özönder Ünal, Cetin Ordu

**Affiliations:** 1 Department of Psychiatry, Tuzla State Hospital, Içmeler Mahallesi, Piri Reis Caddesi, No: 74 Tuzla, Istanbul 34947, Turkey; 2 Division of Medical Oncology, Department of Internal Medicine, Gayrettepe Florence Nightingale Hospital, Cemil Aslan Güder Sk. No: 8, Beşiktaş, Istanbul 34349, Turkey; cetin.ordu@demiroglu.bilim.edu.tr

**Keywords:** cancer, caregiver burden, coping strategies, depression, rumination

## Abstract

This study aimed to elucidate the role of psychological factors in caregiver burden among caregivers of stage 4 cancer patients. Data were collected from 328 caregivers of cancer patients, employing the Zarit Care Burden Scale, Depression-Anxiety-Stress Scale (DASS-42), Dysfunctional Attitudes Scale (DAS-A), Ruminative Thought Style Questionnaire (RTSQ), and Coping Orientation to Problems Experienced Inventory (Brief COPE). Males, spouses, and caregivers of patients with a PEG or tracheostomy, or those diagnosed with pancreatic biliary cancer were found to have a significantly higher risk of caregiver burden. Age, sex, caregiver-patient relationship, caregiving duration, patient’s catheter status, cancer types, depression and stress severity, rumination, dysfunctional attitudes, and dysfunctional coping strategies explained 69.7% of the variance in Zarit Care Burden Scale scores (F(14,313) = 51.457, *p* < 0.001), illustrating their significant predictive relationship with caregiver burden. Moderation analysis revealed significant interactions of emotional coping with depression (b = −0.0524, *p* = 0.0076) and dysfunctional coping with stress on caregiver burden (b = 0.014, *p* = 0.006). Furthermore, rumination mediated the relationships between caregiver burden, stress, and depression (*p* < 0.01). Overall, the results highlight the intricate relationships among caregiver burden, mental health, and coping strategies, suggesting tailored interventions to support caregiver health and quality of care.

## 1. Introduction

Cancer significantly contributes to global disease burden and stands as the second most common cause of mortality in the United States. The lifetime risk of developing cancer in the country is approximately 40.1% for men and 38.7% for women [1]. Its impact in developing nations is also substantial and demands attention. For instance, in Turkey, where this study was conducted, cancer was the cause of an estimated 233,834 new cases and 126,335 deaths in 2020 according to Globocan Turkey [2]. Notably, cancer accounts for 20% of deaths in Turkey, following closely after cardiovascular diseases [3]. It is well documented that caregiving can be a demanding task, with physical, emotional, and financial implications [4]. In this context, caregivers of cancer patients bear a unique and significant burden due to the complexity and unpredictability of the disease trajectory [5].

Existing literature extensively discusses the burden caregivers endure while providing care for a chronically ill loved one, especially cancer patients [4,5,6]. The trajectory of cancer not only impacts patients but also their caregivers, leading to distress and burden. This caregiver burden involves emotional and physical health challenges when caregiving demands surpass resources. Such challenges, varying across different cancer phases, can significantly impact caregiver’s functionality and quality of life. Notably, nearly half of advanced cancer patient caregivers show distress symptoms like depression, anxiety, insomnia, and decreased quality of life [6].

Understanding the determinants and impact of caregiver burden is not just essential; it is imperative in the broader context of holistic healthcare. The well-being of caregivers, who often form the backbone of patient support systems, is paramount, yet they remain a frequently undervalued group [7]. Delving deeper, caregiver burden is an intricate, multifaceted phenomenon. It encompasses a broad spectrum of responses, ranging from physical and psychological to emotional, social, and financial repercussions linked with the caregiving journey [4]. Each of these dimensions plays a pivotal role in shaping the overall experience of a caregiver. Furthermore, as they navigate through the demanding landscape of caregiving, the compounded influence of these myriad stressors frequently manifests in escalated levels of stress, anxiety, and depression. Such negative mental health outcomes do not merely affect caregivers in isolation; they have cascading impacts on the patients they care for and the broader community they interact with [8]. Yet, what amplifies the complexity of this situation is the variability brought about by individual factors. Notably, the nature and degree of the burden felt by caregivers often oscillate based on their unique coping strategies, belief systems, and attitudes. Such personal traits and mechanisms can either buffer against or exacerbate the stresses of caregiving [9]. Recognizing these nuances and understanding their interplay is essential for formulating interventions that truly resonate with caregivers’ needs and challenges.

While the caregiving landscape has been under the academic lens for quite some time, the depth and specificity of studies, especially in the realm of cancer caregiving, leave much to be desired. Despite the plethora of evidence underscoring the profound impact caregiving can have on mental well-being, there exists a noticeable gap in comprehensive studies delving into the intricacies of this field. This oversight is even more pronounced when one considers the multifaceted challenges cancer caregivers often confront [10]. Interestingly, the predominant focus of existing research tends to gravitate towards the adverse psychological ramifications of caregiving, casting a shadow over the brighter aspects of the narrative. Notably, the resilience exhibited by caregivers, their adaptive strategies, and the instances where caregiving nurtures personal growth remain relatively underexplored. While the negative psychological effects certainly warrant attention, there is an equally pressing need to shine a light on the positive facets, such as effective coping strategies and the transformative journey many caregivers undergo [10]. Moreover, a significant dimension that remains relatively untapped is the pivotal role of coping attitudes. These attitudes, which range from resilience and optimism to despair and resignation, play a central role in determining how caregivers adjust to their demanding roles. Their influence does not end there; such attitudes can substantially modulate the extent of caregiver burden, either amplifying or alleviating it. Both empirical evidence and anecdotal accounts suggest the profound implications of these coping mechanisms [11,12]. Hence, they undoubtedly merit a more in-depth exploration, opening avenues for not just academic enrichment but also the development of targeted interventions that can truly make a difference in the lives of caregivers.

The intricate dynamics of caregiving reverberate not only in the individual psyche of the caregiver but also cascade into broader spheres of impact. The well-being of caregivers is not an isolated concern. Instead, it weaves into a larger tapestry, influencing both their personal health trajectories and those of the patients under their care. An emotionally distressed caregiver, grappling with their internal tempests, might inadvertently let these ripple effects permeate the patient’s healthscape. For instance, a caregiver’s emotional turmoil can have tangible repercussions on a patient’s mental equilibrium. This interplay of emotions can manifest in various ways: heightened anxiety levels in the patient, diminished morale, or even spiraling into depressive states. These psychological strains do not operate in a vacuum. They can profoundly affect the physical health of the patient, causing physiological stress responses and exacerbating existing health challenges. Beyond the emotional and physical implications, there is an even more pressing concern—the potential alteration in the patient’s treatment trajectory. Emotional distress in caregivers can lead to gaps in patient care, translating to reduced treatment compliance. Such disruptions can adversely impact the quality of life for the patient, and in severe scenarios, may even amplify mortality risks. This intricate interplay underscores a fundamental truth: caregiving is a symbiotic relationship. The health and well-being of the caregiver and the patient are intertwined, each influencing the other in profound ways. Therefore, addressing the psychological challenges faced by caregivers is not just a matter of ensuring their well-being; it becomes an imperative to safeguard optimal patient care. By enhancing caregiver support, we are not just fostering individual well-being, but we are also paving the way for better patient treatment outcomes and improved quality of life [13].

With this background, we aimed to investigate the relationship between caregivers’ coping attitudes and their levels of care burden, depression, anxiety, stress, and ruminative thinking. Our exploration further delved into understanding the indirect pathways where rumination might serve as a mediating factor. Moreover, a critical facet of our study was to assess how emotional coping and dysfunctional coping mechanisms potentially influence these relationships. In light of these aims, our investigation was designed to provide a comprehensive understanding of how caregivers’ coping strategies might influence their experienced levels of care burden. Alongside this primary objective, we ventured to unravel the interconnectedness of these coping mechanisms with critical indicators of well-being, such as depression, anxiety, and stress.

## 2. Materials and Methods

### 2.1. Study Design and Participants

In this cross-sectional study, the participants included primary caregivers who had been providing care for a family member diagnosed with cancer for at least six months. Aiming to ensure a more homogenous population for our study, this study included those who provide care for family members diagnosed with stage 4 solid tumors. It is important to note that we have deliberately chosen to exclude caregivers of patients suffering from glioblastoma and hematological malignancies. This strategic exclusion is a reflection of the unique complexities associated with these specific types of cancers, which may confound this study’s results.

This study included individuals who are between the ages of 18 and 65 years old. These individuals must be primary caregivers to a relative who has been diagnosed with cancer. The caregiver’s role should involve assisting with the patient’s daily functions, overseeing their medical follow-up and treatment, and addressing their needs. We specifically look for caregivers who are family members and not professional caregivers. Furthermore, these individuals must voluntarily agree to participate in this study, provide informed consent, and be literate.

Individuals diagnosed with psychotic disorders, active mood episodes, or mental retardation were excluded. Also excluded are those with dementia and/or other organic mental disorders. Finally, anyone who does not provide consent or does not meet all the inclusion criteria will be excluded from participating in this study.

A pivotal variable assessed among the caregivers was the duration of care provided each week. This variable quantified the hours dedicated to caregiving tasks, which encompass activities such as assisting with personal care routines (e.g., bathing, feeding), managing medication schedules, coordinating medical appointments, and offering emotional support. Caregivers were classified into two categories based on their weekly caregiving duration:

Less than 10 h/week: This category included caregivers who reported dedicating fewer than 10 h weekly to caregiving. Such caregivers may be assisting patients with milder symptoms or might be sharing the caregiving role with others.

More than 10 h/week: Caregivers in this category are those who commit over 10 h per week to caregiving. This suggests a more intensive caregiving involvement, potentially due to the patient’s advanced disease stage, heightened symptom severity, or lack of additional caregiving support. The distinction at 10 h weekly serves as a meaningful boundary, differentiating between low to moderate and high-intensity caregiving. The intensity of caregiving can notably affect the strain and burden experienced by the caregiver [14].

### 2.2. Data Collection

The caregiving data from 328 patients diagnosed with cancer who have clinical follow-up at the Medical Oncology unit have been considered. Data were collected from December 2022 to June 2023 using a sociodemographic data form, Zarit Care Burden Scale, Depression-Anxiety-Stress Scale (DASS-42), Dysfunctional Attitudes Scale (DAS-A), The Ruminative Thought Style Questionnaire (RTSQ), and the Coping Orientation to Problems Experienced Inventory (Brief COPE). Prior to data collection, participants have received a thorough briefing regarding this study’s objectives, procedures, potential benefits, and risks, followed by obtaining their written informed consent.

Zarit Care Burden Scale (ZBI): Developed to assess the challenges faced by caregivers of individuals requiring care, this scale encompasses 22 items. Each item is scored on a spectrum from 0 (never) to 4 (almost always). Scores ranging from 0–20 indicate ‘no burden’, 21–40 suggest ‘low care burden’, 41–60 represent ‘moderate care burden’, and scores between 61–88 denote ‘severe care burden’. The Turkish validity and reliability study for this scale has been conducted. Higher scores on the scale indicate an elevated caregiver burden. The internal consistency coefficient (Cronbach’s alpha) of the scale was found to be 0.95, denoting strong reliability [15]. In our study, the Cronbach’s alpha was determined to be 0.92, suggesting a very high level of internal consistency.

Depression-Anxiety-Stress Scale (DASS-42): The DASS-42 is a self-report instrument devised to evaluate three closely related negative emotional states: depression, anxiety, and stress. Comprising 42 items, respondents are asked to rate each item using a 4-point Likert scale, which ranges from 0 to 3. Consequently, the scores for each of the three subscales—depression, anxiety, and stress—can span from 0 to 42. It is worth noting that higher scores on any of these subscales signify a greater severity of symptoms associated with that specific emotional state. The Turkish adaptation of the scale has been previously validated and proven to be reliable [16]. For the DASS-depression scale, a cut-off point is established at a score equal to or higher than 10. This threshold corresponds to a sensitivity of 71% and a specificity of 80%. For the DASS-anxiety scale, the cut-off is defined at scores higher than 7. This value aligns with a sensitivity of 88% and a specificity of 56%. Regarding the DASS-stress scale, it is noteworthy that no specific cut-off point was highlighted in this study [16]. For the Turkish version, Cronbach’s alphas were recorded as 0.92 for depression, 0.86 for anxiety, and 0.88 for stress. Notably, in our study, the internal consistency coefficients for the sub-dimensions were slightly different. We found them to be 0.82 for the anxiety sub-dimension, 0.89 for the depression sub-dimension, and 0.92 for the stress sub-dimension. These figures confirm the strong reliability and internal consistency of the DASS-42 sub-dimensions within our sample.

Dysfunctional Attitudes Scale (DAS-A): Based on Beck’s cognitive theory of depression, this scale evaluates dysfunctional assumptions and beliefs. It consists of 40 items, each statement being scored between 1 (totally disagree) and 7 (totally agree), and it is self-rated by the individual. High scores obtained from the scale indicate the frequency of dysfunctional attitudes of the individual. The adaptation, validity, and reliability studies of the scale in the Turkish sample were carried out by Şahin and Şahin [17]. The internal consistency of the Turkish version is good (Cronbach’s α = 0.79) [18]. In our study we found Cronbach’s α = 0.72.

Coping Orientation to Problems Experienced Inventory (Brief COPE): This is the short form of the Coping Orientation to Problems Experienced (COPE) scale that measures different behaviors of people in response to stress. The short form consists of 28 items and 14 subscales. The strategies of acceptance, emotional social support, humor, positive reframing, and religion are categorized as emotion focused. On the other hand, active coping, instrumental support, and planning are considered as problem-focused strategies. Finally, behavioral disengagement, denial, self-distraction, self-blaming, substance use, and venting are considered as dysfunctional coping strategies. The scale was adapted to the Turkish language by Bacanlı et al. [19]. In the validation study conducted in Turkey, the reliability coefficients for various dimensions were as follows: instrumental social support was 0.78, humor was 0.92, focusing and expressing emotions was 0.70, substance and alcohol use was 0.84, acceptance was 0.56, quitting other activities was 0.50, tending toward religion was 0.90, denial was 0.69, behavioral disinterest was 0.59, mental disinterest was 0.62, self-limitation was 0.39, positive reinterpretation was 0.76, using emotional social support was 0.85, and planning was 0.70. In our study, the Cronbach’s alpha coefficients for the subscales were found to be 0.73 for emotion-focused, 0.82 for problem-focused, and 0.64 for dysfunctional coping strategies.

The Ruminative Thought Style Questionnaire (RTSQ) is a 20-item Likert-type scale. Participants score each item based on how well the statement describes them, from 1 (does not describe me at all) to 7 (describes me very well). It aims to assess a general ruminative response style. The validity and reliability of the Turkish version were previously carried out [20]. For the Turkish version, the reliability analysis produced a Cronbach’s Alpha coefficient of 0.91. In the present study, the Cronbach’s Alpha value was measured at 0.86.

### 2.3. Ethical Considerations

The Ethics Committee of Istanbul Bilgi University (protocol code 2022-40162-155 and date of approval 7 December 2022) approved this study. It is performed in accordance with the Declaration of Helsinki. All patients provided written informed consent.

### 2.4. Statistical Analysis

Data were analyzed using SPSS v22 (Statistical Package for Social Sciences). The normality of data was assessed by skewness, kurtosis, and the Kolmogorov-Smirnov test. For the objectives of our study, we found it more informative to utilize the scale scores as continuous variables. This approach allowed us to capture the full spectrum of emotional experiences and avoid potential information loss that could arise from categorizing patients based on cut-off points. By analyzing the scale scores continuously, we could preserve the granularity of the data and explore subtle nuances and variations in emotional well-being among our participants. Independent *t*-test, Pearson’s correlation, ANOVA, or Kruskal-Wallis test were used as appropriate. Univariate and multivariate regression analyses identified predictors of caregiver burden. In regression analysis, precision necessitates the conversion of nominal or categorical variables into numerical form. This was achieved through dummy coding. For instance, “Male” was coded as 0 and “Female” was coded as 1 for sex. Those with a college degree or PhD were coded as 1, whereas all other education levels were designated as 0. When looking at the relation to the patient, “Spouse” was allocated a 1, with other relationships coded as 0. The number of hours of care provision per week was split with less than 10 h per week given a 0, and 10 h or more given a 1. In cancer patient features, patients with a catheter were coded as 1 for “yes”, and 0 for “no”. Finally, in terms of cancer type, pancreaticobiliary cancer was distinguished with a 1, while all other types received a 0. These numerical codes then allowed for smooth integration of these categories into our regression models. Utilizing the PROCESS macro for SPSS [21,22], we systematically implemented two moderation and two mediation models to elucidate the relationships among depression, stress, emotional coping, dysfunctional coping, rumination, and caregiver’s burden as measured by Zarit’s Burden Scale. The essence of our moderation analysis lies in evaluating how a relationship between an independent and a dependent variable may vary under the influence of a third variable, known as the moderator. Consequently, the strength or direction of the primary relationship could shift based on different levels of this moderator. Aligning with our study’s objectives, we integrated two moderation models (Hayes Model 1). The first model delved into the potential alterations in the relationship between depression and caregiver’s burden brought about by emotional coping mechanisms. We hypothesized that these emotional coping strategies could either amplify or diminish the association between heightened depressive symptoms and caregiver’s burden. The second model focused on understanding the potential modulation of the stress-caregiver burden relationship by dysfunctional coping strategies, with our underlying assumption being that such strategies might either enhance or dampen the effect of stress on the caregiver’s perceived burden. Transitioning to mediation, by employing Hayes Model 4, we sought to clarify the direct relationships between depression, stress, and caregiver’s burden. Furthermore, we probed into the indirect pathways influenced by rumination and assessed the interplay of emotional and dysfunctional coping mechanisms on these established relationships. Significance level was set at *p* < 0.05.

## 3. Results

This study engaged 328 caregivers whereupon descriptive characteristics of this study population are presented in Table 1.

In terms of age, the female caregivers (n = 196) had a mean age of 42.71 ± 11.33 years, while male caregivers (n = 132) had a mean age of 44.01 ± 10.01 years; however, the difference was not statistically significant (*p* = 0.277).

**Figure 1:**
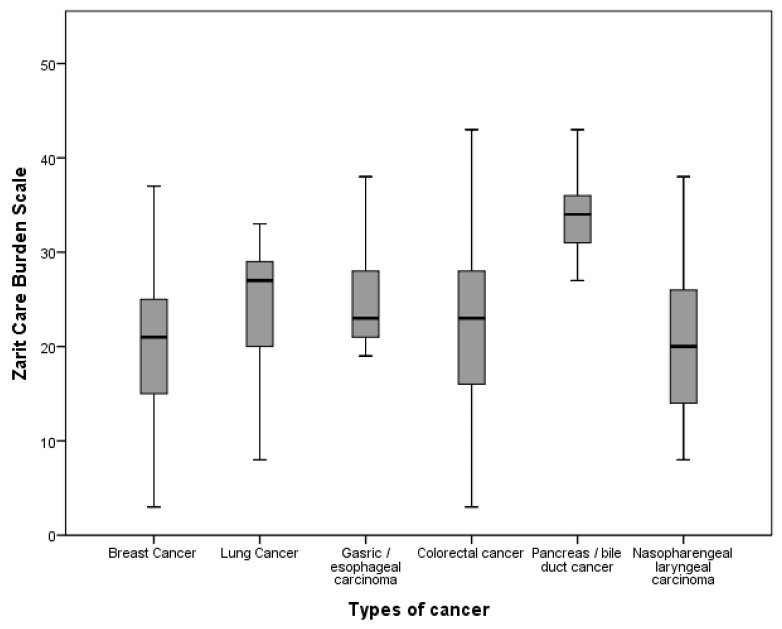
Mean Zarit Burden Scale Scores According to Cancer Types.

Significant differences in Zarit Care Burden Scale scores were observed across categories of sex, education, relationship to patient, number of hours of care provision per week, cancer type, and catheter use (*p* < 0.05) (Figure 1, Table 1). Positive correlations were found with the Dysfunctional Attitudes Scale (r = 0.117, *p* = 0.034), DASS-42 depression (r = 0.577, *p* < 0.0001), stress (r = 0.679, *p* < 0.0001), and Ruminative Thought Style Questionnaire (r = 0.653, *p* < 0.0001), indicating higher scores in these measures associate with higher burden.

The regression model incorporating age, sex, relationship to patient, number of hours of care provision per week, patient’s catheter status, cancer type, DASS-42 Depression, Stress, RTSQ, DAS-A, and dysfunctional coping strategies as independent variables explained 69.7% of the variance in Zarit Care Burden Scale scores (F(14,313) = 51.457, *p* < 0.001). All independent variables, except for education, DASS-42 Anxiety, and emotion-focused coping strategies, significantly predicted Zarit Care Burden Scale scores (Table 2).

For the purpose of this study, two moderation and two mediation models were systematically implemented to unravel the intricate relationships between depression, stress, emotional coping, dysfunctional coping, rumination, and caregiver’s burden, as quantified by Zarit’s Burden Scale.

The first moderation model delved into the role of emotional coping mechanisms as a possible moderator between depression and caregiver’s burden. The analysis revealed a significant direct effect between depression and caregiver’s burden (b = 3.14, CI = 2.79 to 3.49, *p* < 0.0001), which means that an increase in depression is significantly correlated with an increase in caregiver’s burden. Furthermore, the moderating effect of emotional coping mechanisms on this relationship was also significant (b = −0.0524, CI = −0.0917 to −0.0131, *p* = 0.0076).

The second moderation model examined the moderating role of dysfunctional coping strategies on the relationship between stress and caregiver’s burden. The results showed that stress has a significant direct effect on caregiver’s burden (b = 0.3888, CI = 0.0797 to 0.6980, *p* = 0.0138). Meaning, as stress levels rise, so does the caregiver’s burden. The moderating effect of dysfunctional coping strategies was statistically significant (b = 0.0142, CI = 0.0041 to 0.0242, *p* = 0.0060).

Shifting our focus to the mediation models, it is pivotal to comprehend the underlying principles of mediation analysis. This analytical method illuminates the indirect influence that an independent variable (IV) might have on a dependent variable (DV) by routing it through a mediator (M). The essence of mediation analysis is to elucidate the mechanism or process that drives an observed relationship between the IV and DV, with the mediator serving as the explanatory intermediary.

In this study context, we employed Hayes’ Model 4 to conduct two distinct mediation analyses, each aiming to elucidate a simple mediation process.

Model A (Stress-Rumination-Care Burden): This model delves into the mediating role of rumination between stress (the independent variable) and caregiver burden (the dependent variable). The model’s integral components include:

Effect of Stress on Rumination (Stress -> Rumination): Measures the influence of stress on rumination levels.

Effect of Stress on Care Burden (Stress -> Care Burden): Quantifies the immediate influence of stress on caregiver burden without the mediator’s interplay.

Effect of Rumination on Care Burden (Rumination -> Care Burden): Assesses how rumination translates into caregiver burden.

Indirect Effect (Stress -> Rumination -> Care Burden): Represents the mediating influence, signifying how stress impacts caregiver burden through the medium of rumination.

Model B (Depression-Rumination-Care Burden): Mirroring Model A in structure, this model spotlights rumination’s mediating role between depression (the independent variable) and caregiver burden (the dependent variable). All components align with those of Model A, but here the focal point shifts from stress to depression.

**Figure 2:**
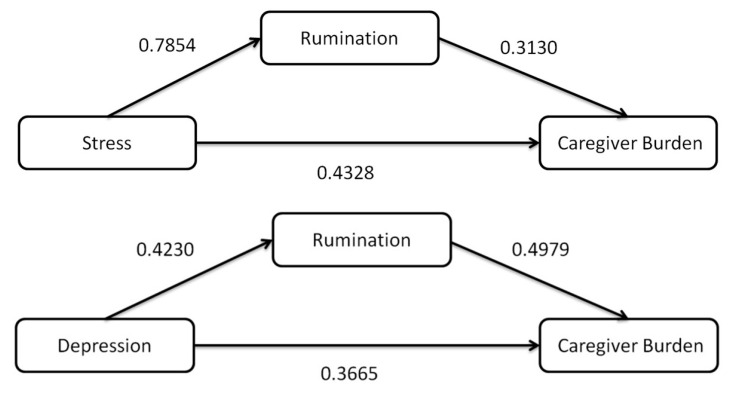
Structural models with hypothesized links between stress, depression, and caregiver burden with rumination as mediators. Values are for standardized coefficients.

The first analysis explored the role of rumination as a mediator in the relationship between stress and caregiver’s burden. The analysis found a significant direct effect of stress on caregiver’s burden (b = 0.5186, CI = 0.4278 to 0.6094, *p* < 0.0001), illustrating that increasing stress levels are associated with a higher caregiver’s burden. Rumination was found to partially mediate this relationship (Effect = 0.2946, CI 0.0726 to 0.5530) (Figure 2, Table 3).

The second mediation analysis examined the mediating role of rumination in the relationship between depression and caregiver’s burden. The results revealed a significant direct effect of depression on caregiver’s burden (b = 1.0485, CI = 0.8139 to 1.2830, *p* < 0.0001), indicating that higher depression levels correspond with increased caregiver’s burden. The indirect effect of depression on caregiver’s burden through rumination was also significant (Effect = 0.6026, CI 0.3175 to 1.0183), further solidifying the role of rumination as a significant mediator in this relationship (Figure 2, Table 3).

## 4. Discussion

This study delves into the multifaceted dynamics of caregiver burden among those tending to cancer patients. Key findings indicated a heavier burden borne by highly educated, younger caregivers and those caring for a spouse. Caregiving tasks and the nature of the patient’s cancer significantly influenced the burden. Specifically, emotional coping strategies were found to moderate the effect of depression on caregiver burden, whereas dysfunctional coping strategies influenced the relationship between stress and caregiver burden. Furthermore, rumination was identified as a mediator in the relationships between both stress and depression and caregiver burden. These findings provide valuable insights into the multifaceted dynamics of caregiver burden in a cancer care context.

Our study’s findings concerning caregiver burden among cancer patients, using the Zarit Care Burden Scale (ZBI), elucidate the multifaceted nature of caregiver experiences across different conditions. Considering our study did not incorporate a control group, consulting the broader literature for context is essential. In the realm of medical research, caregiver burden has been a focal point of several studies due to its impact on the mental, emotional, and physical well-being of caregivers. The Zarit Burden Interview (ZBI) scores provide quantifiable insights into these challenges across different studies. Research has spanned conditions from Alzheimer’s to congestive heart failure, revealing the profound impacts of caregiving across a myriad of medical scenarios. For instance, a study on caregivers of Alzheimer’s disease patients by S. Liu et al. in China reported a mean ZBI score of 12.2 ± 13.2, unveiling significant challenges faced by these caregivers, including depression, anxiety, and sleep disturbances [23]. Similarly, caregivers of patients with congestive heart failure, as studied by P.J. Hooley et al. in 2005, showcased a ZBI score of 16.0 ± 14.4, shedding light on the intertwined relationship between caregiver depressive symptoms and the patient’s illness severity [24]. In the realm of Parkinson’s, a study by Vatter et al. in 2018 reported an average ZBI score of 35.51, highlighting the complexities of caregiving for patients with Parkinson-related dementia [25]. Further diversifying the spectrum, caregivers for liver transplant patients and children diagnosed with cancer reported average caregiving burden scores of 33.77 and 21.29, respectively, adding depth to our understanding of caregiver burdens in varied conditions [26,27].

Transitioning specifically to cancer caregiving, it is noteworthy how the dynamics and challenges differ from other medical conditions. A compelling study by P. Hu et al. in 2018 observed caregivers of stroke patients and revealed a mean ZBI score of 25.88 ± 10.35, underscoring the profound connection between anxiety, depression, and care burden [28]. Breast cancer caregiving was spotlighted by Y. Li et al. in 2018 and Vahidi et al. in 2016, with mean ZBI scores of 24.8 ± 12.5 and 30.55 ± 19.18, respectively, providing insights into the role of family and individual resilience as well as influential factors like daily living activities and caregiver education [29,30]. J. Liu’s 2021 research on Chinese American dementia caregivers reported a significant range in ZBI scores, with a mean score of 40.35 ± 16.53, offering a unique demographic perspective [31]. Lastly, Harding et al.’s 2015 comparative analysis of caregiver burden across patient groups reported cancer caregivers with a ZBI score of 23.3, showcasing the distinctive challenges they face [32]. From these diverse studies, it becomes evident that the mean care burden scores fluctuate based on the specific condition, cultural background, and other influencing factors. Addressing caregiver burden requires a multifaceted approach tailored to these distinct parameters.

Our study underscores male sex and younger age as significant factors associated with higher caregiver burden. The increased burden among male caregivers can be ascribed to traditionally defined gender roles, a lack of caregiving experience and training, less expressive emotional coping mechanisms, and fewer social support resources than women. Further, men might experience a heightened burden due to more pronounced health challenges and potentially less effective problem-focused coping strategies when managing the immutable stressors inherent in caregiving. In line with the literature, younger caregivers experiencing greater burden highlights a need for targeted interventions for this demographic [33]. The higher burden among younger caregivers indicates a need for interventions aimed specifically at this demographic, who may be concurrently managing caregiving with other demanding responsibilities such as employment or raising children.

Contrary to a review [33], our findings did not show a significant difference in caregiver burden relative to employment status. Interestingly, we found that higher education, as opposed to primary or lower education, was linked with an increased burden among caregivers of cancer patients. Our results showed that prolonged education correlated with a higher intensity of caregiver burden. While some studies propose that a higher level of education results in better coping skills and, therefore, a reduction in caregiver burden [33], others suggest that caregiver burden is higher among university graduates [34]. This discrepancy could be attributed to several factors. Firstly, higher education is often linked to higher life standards and expectations, which when unmet due to caregiving demands, can contribute to feelings of burden. Furthermore, those with higher education may also have demanding professional responsibilities, which, when combined with the stress and time commitment of caregiving, compound the burden [33].

Our study confirmed the association between the caregiver’s relationship to the patient and the caregiver burden, echoing findings by Ge and Mordiffi [33], where spouses were found to bear a higher burden. This reaffirms the emotional and psychological impact of caregiving for a critically ill partner. Notably, we did not specifically find a higher burden among sibling or child caregivers as suggested by the review [33].

The significant caregiver burden associated with caring for patients with rapidly progressing cancers, notably pancreatic cancer or bile duct tumors, is due to the demanding care tasks and emotional strain induced by the swift deterioration of the patient’s health [35]. Moreover, caregivers of pancreatic cancer patients often need to provide varying levels of support, ranging from emotional care to practical assistance, like contributing to treatment decisions, attending medical appointments, and coordinating communication among the medical team and family members [36,37].

On the other hand, caregivers of breast cancer patients exhibited the lowest burden scores, potentially due to several factors. Given that breast cancer is the most common cancer worldwide, there may be greater social awareness, support resources, and research on coping mechanisms specifically for this cancer type. This, in conjunction with other factors such as shared faith, community solidarity, and the collective fight against breast cancer, could contribute to lower perceived caregiver burden in this group. Increased public awareness and support for less common, but equally devastating cancers, could be beneficial in aiding caregivers in these areas [38].

Furthermore, this study highlights that an extended duration of caregiving (more than 10 h a week) and caring for patients with specific medical procedures like percutaneous endoscopic gastrostomy (PEG) or tracheostomy significantly contribute to caregiver burden. These caregiving tasks require considerable time commitment, specialized skills, and may cause emotional strain due to their invasive nature and association with severe disease states.

We hypothesized that levels of depression, anxiety, and stress, along with coping attitudes and ruminative thinking styles, are interconnected and could predict caregiver burden. Indeed, as anticipated, factors such as caregiver’s age, sex, relationship to the patient, number of hours of care provision per week, patient’s catheter status, cancer types, levels of depression and stress, ruminative thinking style, and dysfunctional coping strategies were all found to significantly predict the level of caregiver burden.

Additionally, we sought to uncover any potential moderating and mediating effects these factors might have on caregiver burden. As one of the first studies to undertake such a comprehensive investigation in the context of cancer caregiving, our findings could significantly contribute to our understanding of caregiver burden and aid in developing effective strategies for caregiver support and intervention.

Our study implemented a systematic approach to investigate the complex interplay of psychological and coping factors influencing caregiver burden. Analysis revealed that emotional coping modifies the relationship between depression and caregiver burden, suggesting that effective emotional coping can potentially mitigate the burdening effect of depression. Dysfunctional coping strategies emerged as a critical factor moderating the impact of stress on caregiver burden, hinting that maladaptive coping can exacerbate the burden felt during stressful situations.

Our study found that emotion-focused coping strategies significantly contributed to caregiver burden. On the other hand, Teixeira et al. found that problem-focused coping is linked with reduced caregiver burden [6]. This discrepancy could be due to varying caregiving demands and disease stages.

Whether problem-focused or emotion-focused coping is more beneficial for caregivers is complex, as their effectiveness varies based on multiple factors, including caregiver’s personality, patient’s condition, and specific stressors. Emotion-focused strategies can help manage uncontrollable stressors and maintain emotional balance. For example, social support can provide emotional comfort, and positive reframing can assist in finding positivity amidst adversity. On the other hand, problem-focused strategies, like planning and instrumental support, can be useful when dealing with controllable aspects of caregiving, reducing feelings of being overwhelmed. It may be more beneficial for caregivers to use a mix of both strategies, tailored to their needs. Future research should further examine the relative effectiveness of these strategies in different caregiving contexts, considering caregivers’ mental resilience and the adaptive nature of coping strategies.

Our research identified rumination as a significant mediating factor between stress, depression, and caregiver burden. Rumination, or the persistent dwelling on distressing thoughts, can amplify the impact of stress and depression on caregiver burden, underlining the vital role of psychological factors and coping strategies in managing caregiver burden [39].

Rumination, as continuous fixation on negative events and implications, intensifies caregiver burden by heightening anxiety and negative emotions [40]. It may be triggered by thoughts about the patient’s prognosis, the disease’s progression, or future existential concerns. While sometimes associated with post-traumatic growth, rumination more often leads to an increased focus on problems, culminating in psychological distress. This distress can further compound caregiver burden. Moreover, rumination often encourages avoidant coping strategies, representing a risk factor for higher stress and anxiety levels [39].

The relationship between caregiver burden and depression, complicated by factors like rumination and feelings of hopelessness, is multifaceted. High caregiver burden can precipitate depression, but existing depression could also magnify the perceived caregiver burden. Moreover, rumination, common in depressed individuals, may amplify this perceived burden. While the complexity of these associations necessitates further research, recognizing these intricacies could inform future studies and help refine caregiver support strategies [41].

### 4.1. Limitations

While our study offers significant insights into the complex interplay of mental health, coping mechanisms, and caregiver burden, it has some limitations. One significant constraint is the absence of a control group, which might have provided a more comparative insight. The cross-sectional design prevents us from making definitive causal claims about observed relationships, and a longitudinal design in future research could provide a more dynamic view. The use of self-report measures could introduce bias. A pertinent concern is the sole reliance on self-reported measures, notably the ZBI, which can introduce bias. Especially in societies like Turkey, distinguished by extended family systems, caregiving can adopt a different perspective. In such cultural contexts where joint family living and shared responsibilities are common, caring for a loved one, especially in terminal stages, might not conventionally be voiced or even perceived as a “burden”. Cultural values and collective beliefs can influence caregivers’ responses to such scales. The potential for underreporting could stem from not just an internalized sense of duty but also prevailing societal opinions. Expressing feelings of distress or being overwhelmed might be construed as a weakness, leading caregivers to fear criticisms for possibly not upholding family duties. Instruments like the ZBI, while valuable, often miss these subtleties. Our focus on caregivers of stage 4 cancer patients limits the generalizability of our findings for caregivers of patients in other cancer stages, while it strengthens the relevance of this study for this particular group. We also did not consider potential confounding variables such as caregivers’ physical health, social support, and financial strain. This study was conducted in a single geographical location, potentially limiting the generalizability of the findings to other regions or cultures with differing healthcare systems and cultural attitudes.

### 4.2. Clinical Implications

This study illuminates significant clinical implications, particularly highlighting key at-risk groups and potential intervention targets. A higher risk of caregiver burden was discerned among males, individuals with a higher level of education, spouses, and caregivers tending to patients with a percutaneous endoscopic gastrostomy (PEG) or tracheostomy, or a diagnosis of pancreatic biliary cancer with rapid deterioration. These findings underscore the urgent need for healthcare professionals to be especially vigilant with these caregiver demographics, ensuring they receive necessary support and mental health screenings. Furthermore, our investigation brings to the fore the mediating and moderating roles of rumination, dysfunctional attitudes, and emotion-focused coping strategies in caregiver burden. By focusing interventions on these psychological mechanisms, we might be able to lessen caregiver burden significantly. For instance, cognitive-behavioral therapy could be tailored to address dysfunctional attitudes and rumination, while emotion-focused coping strategies could be enhanced through stress management programs or mindfulness-based therapies. Our findings thus provide a solid foundation for more targeted, individualized caregiver support, and highlight the need for policymaking to be cognizant of these risk factors and therapeutic avenues.

## 5. Conclusions

This study has elucidated the multifaceted nature of caregiver burden, particularly in caregivers for patients with stage 4 cancer. We found that higher levels of stress and depression among caregivers were significantly associated with greater caregiver burden. Furthermore, our data highlighted the critical mediating role of rumination and the moderating role of emotional and dysfunctional coping strategies in this relationship.

In terms of risk groups, we identified several key demographics at heightened risk of experiencing substantial caregiver burden. The complexity of these findings underscores the necessity for an individualized, multifaceted approach to supporting caregivers. This involves not only addressing their psychological wellbeing through interventions targeting rumination, dysfunctional attitudes, and coping strategies but also ensuring that these interventions are tailored to their specific demographic and situational needs.

By shedding light on the interplay between various factors and caregiver burden, our research paves the way for future studies, spurring the creation of finely tuned interventions geared towards bolstering the mental resilience of caregivers and diminishing the weight of caregiver burden. We advocate for subsequent longitudinal research, encompassing broader and more varied caregiver cohorts, to both validate and enrich our findings on this critical subject.

## Figures and Tables

**Table 1:** Demographic and Clinical Characteristics of Caregivers by Zarit Care Burden Scale Scores in Cancer Patients.

	n (%)	Zarit Care Burden Scale Scores	*p* Value
Age (mean ± Standart Deviation)	43.2 ± 10.8		
**Sex**			0.008 ^a^
female	196 (59.8)	22.1 ± 6.8	
male	132 (40.2)	24.6 ± 8.9	
**Education**			0.006 ^b^
primary/secondary school	105 (32.0)	21.9 ± 6.5	
high school	159 (48.5)	22.8 ± 8.1	
college/Ph D	64 (19.5)	25.9 ± 8.5	
**Relationship to the patient**			0.003 ^b^
sibling	87 (26.5)	21.4 ± 6.4	
spouse	145 (44.2)	24.7 ± 8.8	
children	96 (29.3)	22.1 ± 6.9	
**Number of h of care provision per week**			0.006 ^a^
less than 10 h/week	121 (36.9)	21.6 ± 7.0	
more than 10 h/week	207 (63.1)	24.1 ± 8.2	
**Types of cancer**			<0.001 ^c^
Breast cancer	122 (37.2)	20.5 ± 7.4	
Lung cancer	65 (19.8)	23.92 ± 6.3	
Gasric/Esophageal Carcinoma	33 (10.1)	25.2 ± 4.9	
Colorectal cancer	62 (18.9)	22.7 ± 8.4	
Pancreas/Bile duct cancer	25 (7.6)	33.5 ± 4.0	
Nasopharengeal Laryngeal carcinoma	21 (6.4)	21.4 ± 8.6	
**Patient having a catheter**			0.01 ^a^
Yes	37 (11.3)	26.2 ± 7.6	
No	291 (88.7)	22.7 ± 8.7	

Independent samples *t*-test ^a^, one-way ANOVA ^b^, Kruskal-Wallis test ^c^.

**Table 2:** Univariate and Multivariate Regression Analysis of Factors Influencing Zarit Care Burden Scale Scores in Cancer Caregivers.

	Unstandardized				Standardized	
	B	SE	Lower	Upper	β	*p* Value
*Univariate regression analysis*						
age	−0.10	0.04	−0.18	−0.02	−0.13	0.01
Sex(male vs. female)	2.45	0.87	0.73	4.16	0.15	0.005
education (college/PhD vs. lower)	3.50	1.07	1.39	5.61	0.17	0.001
Relation to the patient (spouse vs. others)	2.94	0.86	1.25	4.62	0.18	0.001
Number of hours of care provision per week (≥10 h per week vs. <10 h per week)	2.46	0.88	0.72	4.21	0.15	0.006
Cancer patient features						
Patient having a catheter (yes vs. no)	3.50	1.35	0.84	6.17	0.14	0.010
Types of cancer (pancreaticobiliary cancer vs. others)	11.27	1.50	8.31	14.24	0.38	<0.001
Scale Scores						
DASS-42 Depression	1.65	0.12	1.39	1.90	0.57	<0.001
DASS-42 Anxiety	0.39	0.19	0.01	0.78	0.11	0.04
DASS-42 Stress	0.81	0.04	0.71	0.90	0.67	<0.001
Ruminative Thought Style Questionnaire (RTSQ)	0.29	0.01	0.25	0.33	0.65	<0.001
DAS-A	0.02	0.01	0.01	0.05	0.11	0.03
Brief-COPE						
Problem focused coping strategies	−0.12	0.11	−0.34	0.09	−0.06	0.25
Emotion focused coping strategies	−0.18	0.06	−0.31	−0.05	−0.15	0.006
Dysfunctional coping strategies	0.27	0.05	0.16	0.37	0.27	<0.001
*Multivariate regression analysis*						
age	−0.07	0.02	−0.13	−0.02	−0.11	0.002
Sex (male vs. female)	1.54	0.60	0.35	2.72	0.09	0.01
education (college/PhD vs. lower)	0.71	0.64	−0.55	1.97	0.03	0.27
Relation to the patient (spouse vs. others)	2.73	0.78	1.19	4.28	0.17	0.001
Number of hours of care provision per week (≥10 h per week vs. <10 h per week)	−2.35	0.74	−3.81	−0.89	−0.15	0.002
Cancer patient features						
Patient having a catheter (yes vs. no)	3.34	0.84	1.68	5.00	0.13	<0.001
Types of cancer (pancreaticobiliary cancer vs. others)	6.81	0.98	4.87	8.74	0.23	<0.001
Scale Scores						
DASS-42 Depression	0.74	0.10	0.52	0.95	0.26	<0.001
DASS-42 Anxiety	0.12	0.11	−0.09	0.35	0.04	0.27
DASS-42 Stress	0.29	0.06	0.16	0.43	0.25	<0.001
Ruminative Thought Style Questionnaire (RTSQ)	0.12	0.02	0.07	0.17	0.27	<0.001
DAS-A	0.02	0.01	0.01	0.04	0.11	<0.001
Brief-COPE						
Emotion focused coping strategies	−0.01	0.04	−0.09	0.07	−0.01	0.77
Dysfunctional coping strategies	0.10	0.03	0.03	0.18	0.11	0.005

This table displays unstandardized (B) and standardized (β) regression coefficients for both univariate and multivariate regression analyses (Linear Regression), along with the standard errors (SE), 95% confidence intervals (Lower, Upper), and *p*-values. Depression-Anxiety-Stress Scale (DASS-42), Dysfunctional Attitudes Scale (DAS-A), and Coping Orientation to Problems Experienced Inventory (Brief COPE).

**Table 3:** Results of Mediation Analysis (Hayes Model 4): Effects of Stress and Depression on Caregiver Burden via Rumination.

	Effect	Standardized Error	Standardized Coeffcient	t	Lower	Upper	*p* Value
***Model A: Stress -> Rumination -> Care Burden***							
***Components***							
Stress -> Rumination	2.06	0.09	0.78	22.60	1.89	2.24	<0.001
Stress -> Care Burden	0.51	0.07	0.43	6.81	0.36	0.66	<0.001
Rumination -> Care Burden	0.14	0.02	0.31	4.92	0.08	0.19	<0.001
***Indirect effect***							
Stress -> Rumination -> Care Burden	0.30	0.12	2.24		0.07	0.55	<0.001
***Total effect***							
Stress -> Care Burden	0.81	0.04	6.67	16.68	0.71	0.90	<0.001
***Model B: Depression -> Rumination -> Care Burden***							
***Components***							
Depression -> Rumination	2.66	0.32	0.42	8.42	2.03	3.28	<0.001
Depression -> Care Burden	1.05	0.12	0.36	8.79	0.81	1.28	<0.001
Rumination -> Care Burden	0.22	0.02	0.49	11.94	0.18	0.26	<0.001
***Indirect effect***							
Depression -> Rumination -> Care Burden	0.60	0.18	0.21		0.31	1.01	<0.001
***Total effect***							
Depression -> Care Burden	1.65	0.12	0.57	12.75	1.39	1.90	<0.001

## Data Availability

Data supporting the findings of this study are available upon reasonable request from the corresponding author.
